# Interobserver Agreement and Reference Intervals for Biventricular Myocardial Deformation in Full-Term, Healthy Newborns: A 2D Speckle-Tracking Echocardiography-Based Strain Analysis

**DOI:** 10.3390/ijerph19148620

**Published:** 2022-07-15

**Authors:** Daniela Toma, Rodica Toganel, Amalia Fagarasan, Manuela Cucerea, Dorottya Gabor-Miklosi, Andreea Cerghit-Paler, Diana-Ramona Iurian, Horea Gozar, Elena Moldovan, Mihaela Iancu, Liliana Gozar

**Affiliations:** 1 Department of Pediatric Cardiology, Emergency Institute for Cardiovascular Diseases and Transplantation, 540136 Targu Mures, Romania; tomadaniela94@yahoo.com (D.T.); amalia.fagarasan@umfst.ro (A.F.); annadorka@yahoo.com (D.G.-M.); palerandreea@yahoo.com (A.C.-P.); dianaiurian@yahoo.com (D.-R.I.); lili_gozar@yahoo.com (L.G.); 2 Department of Pediatrics, “George Emil Palade” University of Medicine, Pharmacy, Science and Technology of Targu Mures, 540139 Targu Mures, Romania; rodica.toganel@umfst.ro; 3 Department of Neonatology, County Emergency Hospital Targu Mures, 540136 Targu Mures, Romania; manuela.cucerea@umfst.ro; 4 Department M3, Pediatric IV, “George Emil Palade” University of Medicine, Pharmacy, Science and Technology of Targu Mures, 540139 Targu Mures, Romania; 5 Department of Pediatric Surgery, “George Emil Palade” University of Medicine, Pharmacy, Science and Technology of Targu Mures, 540139 Targu Mures, Romania; horea.gozar@umfst.ro; 6 Pediatric Intensive Care Unit, Emergency Institute for Cardiovascular Diseases and Transplantation, 540136 Targu Mures, Romania; bitirelena@gmail.com; 7 Department of Medical Informatics and Biostatistics, Faculty of Medicine, “Iuliu Hațieganu” University of Medicine and Pharmacy Cluj-Napoca, 400012 Cluj-Napoca, Romania

**Keywords:** myocardial strain, speckle tracking, newborn, echocardiography

## Abstract

Data regarding reference intervals for strain parameters derived from 2D speckle-tracking echocardiography in full-term newborns are limited and still under development. Our objectives were to establish the level of reproducibility and reference intervals in assessing myocardial function using 2D speckle-tracking echocardiography for longitudinal and regional strain measurements. A total of 127 full-term newborns were examined to be included in the study, of which 103 were analyzed. We used two-dimensional acquisitions from apical four-chamber view of both ventricles and analyzed the autostrain function offline. We obtained interobserver agreement between the two observers ranging from good to excellent for all speckle-tracking parameters except for the strain of the medial portion of the left ventricle (LV) lateral wall and the strain measured on the basal portion of the inter-ventricular septum, which reflected a fair interobserver reproducibility (ICC = 0.52, 95% IC: 0.22–0.72 and ICC = 0.43, 95% IC: 0.12–0.67, respectively). The reference values obtained for the LV peak longitudinal strain were between −24.65 and −14.62, those for the right ventricle (RV) free wall were from −28.69 to −10.68, and those for the RV global four-chamber were from −22.30 to −11.37. In conclusion, two-dimensional peak longitudinal LV and RV strains are reproducible with good to excellent agreement and may represent a possible alternative for the cardiac assessment of healthy newborns in the clinical practice.

## 1. Introduction

Assessment of left ventricular function (LV) is of primordial importance in the diagnosis, risk stratification, and management of patients with hemodynamically unstable heart disease. When assessing left ventricular ejection fraction (LVEF), both the European Association of Cardiovascular Imaging (EACVI) and American Society of Echocardiography (ASE) additionally recommend performing the peak longitudinal strain (pGLS). The analysis of LV function through this important parameter is rarely used in the daily clinical practice, as it is considered time-consuming and requires some experience from the examiner [1]. Assessment of LV function by conventional echocardiographic methods, such as the shortening fraction (SF) and ejection fraction (EF), parameters that assess changes in the size of the cavities of the heart, are insufficient in detecting the dysfunction in a timely fashion. Often, these measurements may be influenced by the quality of the images, their reproducibility, and inadequate standardization in newborns. Similarly, conventional methods of estimating right ventricular (RV) performance are largely based on quantitative and qualitative predictions that are limited by the unique three-dimensional structure of the ventricular chamber.

The concept of deformation imaging, a new and recently introduced technique in the field of neonatal cardiology, consists of speckle-tracking echocardiography (STE) or tissue Doppler imaging (TDI). Myocardial strain is defined by tissue deformation, and the strain rate (SR) is the velocity at which the deformation occurs. Both are feasible and reproductible markers of global and regional performance by providing fundamental information on myocardial and mechanical properties, such as early ventricular dysfunction (through the detection of subclinical myocardial dysfunction), data otherwise unavailable by conventional imaging [2,3] . Therefore, strain parameters have been included in most updated guidelines for the echocardiographic assessment of adults. Longitudinal strain is widely used, having shown the best reproducibility in preterm and full-term newborns [4,5,6,7,8,9]. Longitudinal RV deformation is obtained from the four-chamber apical view, with a focus on the RV, the method proving itself to be the most feasible and reproducible analysis of RV deformation in newborns [4,9]. Although in theory, the septum is considered to be bi-stratified, contributing to the function of both ventricles, in the clinical practice, it is considered to be part of the LV. In newborns, the analysis of deformation parameters is performed offline, using specialized software. Although newer imaging systems and software allow strain evaluation directly on the echocardiograph during image acquisition, the feasibility and reliability of this method have not yet been evaluated in newborns [4,9]. Normative data and reference intervals are rare and still under development for strain parameters obtained using STE 2D in premature and full-term newborns. Studies in the literature include a relatively small number of newborns [9].

The present study aims to determine the interobserver reproducibility and reference intervals of longitudinal and regional strains measurements in the left and right ventricle and interventricular septum.

## 2. Materials and Methods

**Figure 1:**
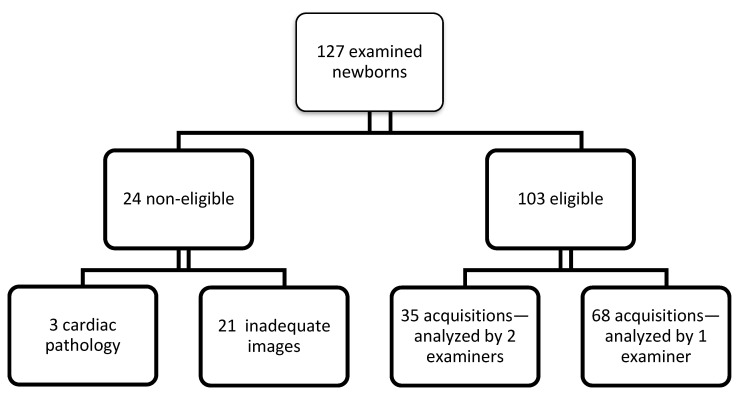
Study flowchart.

The study was conducted for 14 months, more precisely between December 2020—January 2022, at the Cardiology Clinic from the Emergency Institute of Cardiovascular Diseases and Transplantation, Targu Mures. The flow chart of patients’ selection is presented in Figure 1. Inclusion criteria: full-term newborns (gestational age 37–42 weeks), singletons, 1 to 28 days of age, no clinical signs or personal history suggestive of perinatal distress (APGAR score over 8), no associated systemic or cardiac pathologies, no treatment at the time of evaluation, and good-quality apical four-chamber view acquisitions that allow the analysis of all the above-mentioned segments. Exclusion criteria: newborns with perinatal hypoxic distress (APGAR score < 8), premature infants, low birth weight infants for gestational age, as well as those with cardiac pathology or other associated malformations, and recorded cases with unsatisfactory, poor-quality images.

This study was approved by the ethics committees of the Institute of Cardiovascular Diseases and Transplantation and “George Emil Palade” University of Medicine and Pharmacy Targu-Mures 1276/25.02.2021.

The echocardiographic acquisitions of the whole group were initially analyzed by a first investigator, later on, 40 images of the newborns included in the study were analyzed by the second observer.

All echocardiograms were performed using an Epiq 7 ultrasound machine. A complete echocardiographic evaluation was performed to rule out both structural and functional cardiac pathologies. Classic echocardiographic parameters for the assessment of the systolic function included the following: lateral mitral annular plane systolic excursion—MAPSE, lateral tricuspid annular plane systolic excursion—TAPSE, as well as the M mode LVEF Teicholz. Regarding strain parameters, the peak GLS and the biventricular segmental strain were determined, the interventricular septum and the walls being divided into three segments (LV basal, LV medial, LV apical; inter V basal, inter V medial, inter V apical; RV basal, RV medial, RV apical, RV free wall, RV4C). We also mention that our software generates the acronym LVpGLS for the LV peak longitudinal strain and RVFWSL and RV4CSL for RV peak longitudinal strain, and we maintain those as abbreviations herein.

The two-dimensional acquisitions from an apical four-chamber view as well as the four-chamber view centered on the RV were recorded with a frame rate of at least 70 Hz and optimal quality, three-cycle analysis, stored in Digital Imaging and Communications in Medicine (DICOM) format and analyzed offline with the Philips QLAB 15 software, using its LV autostrain and RV autostrain functions, respectively. In order for the analysis to be made on its entire length, the cardiac cycle was defined automatically by the software through the movement of the mitral valve in M-mode. After three points were chosen on the endocardial level (base of the septum, lateral, and apical), the edge of the endocardium was defined at first automatically but later corrected manually. Both echocardiographic acquisitions and speckle-tracking analysis were performed by two examiners, both pediatric cardiologists with extensive experience in the field.

### Statistical Analysis

Demographic and clinical quantitative variables with normal distributions were summarized by the arithmetic mean and standard deviation (SD). Clinical variables with departures from normal distribution were summarized using median with interquartile interval IQR = (25th percentile; 75th percentile). The assessment of normal distribution was performed by the estimation of univariate kurtosis and skewness, Q-Q plot, Shapiro–Wilk, and Anderson–Darling tests. Qualitative clinical characteristics were described using absolute and relative frequencies.

Linear or monotone relationship of regional and peak longitudinal strain with age, gestational age, gender, and birth weight were tested and quantified by Pearson or Spearman’s correlation coefficients, the last method being used for strain measurements that did not follow a normal distribution. Differences concerning longitudinal strain values distributions by diagnostic type were tested using Kruskal–Wallis test followed by a post hoc analysis using Dunn’s test. Statistical significance for all two-sided tests was obtained if the estimated significance level (*p*-value) was ≤0.05.

Inter-rater reproducibility of regional and peak longitudinal strain measurements was assessed using intra-class correlation coefficient ICC (2,1) with their 95% confidence intervals (95% CI) based on two-way random effects models for single-rater agreement. The level of reliability was based on the recent guidelines according to the Bunting classification as follows: a value of ICC < 0.40 denoted poor agreement, an ICC between 0.40 and 0.59 was fair, a value of ICC between 0.60 and 0.74 was indicative of good reliability, and ICC of 0.75–1.00 indicated an excellent agreement [10].

The global agreement between speckle-tracking values between two observers was evaluated by Bland–Altman method used to quantify constant bias or systematic error measurement (as an indicator of lack of agreement). The Bland–Altman plots were used to show the difference in scores of speckle-tracking parameters between two observers against mean scores for each subject and 95% limits of agreement (LOA). In addition, we added the 95% CI of agreement limits (95% CI: Lower CL−Upper CL, where CL = confidence limit) in order to quantify the precision of estimated limits of agreement. We also tested the presence of proportional bias of strain measurements between the two observers using Passing–Bablok regression. The intercept and slope of Passing–Bablok (PB) regression line were estimated with their 95% confidence intervals (CI). If 95% CI of intercept did not contain 0, there was systematic bias for the strain measurements between the two observers. The slope of PB regression was used to quantify the difference in the ratio of strain measurements between the two observers. If the 95% CI for slope did not contain the reference value of 1.00, we concluded that there was not enough evidence to conclude that the methods were not in agreement. The CUSUM test for linearity indicated no significant departure from linearity for all strain measurements.

In order to estimate the reference interval (RI), we tested the presence of outliers in regional and peak strain measurements using Horn’s method applied on Box and Cox transformed data and [11,12] Tukey’s interquartile fences. A point was considered an outlier if it was outside the interval Q1—1.5 × IQR and Q3 + 1.5 × IQR, where Q1 = first quartile, Q3 = third quartile, and with IQR being the interquartile range. RI with and without outlier removal was estimated by the parametric and robust method for Gaussian data distribution and nonparametric method for data with departures from Gaussian distribution. The upper reference limits (UL) and lower reference limits (LL) for RI based on parametric method were calculated at mean + 1.96SD and mean + 1.96SD (SD = sample standard deviation). The robust method involved a transformation of the data according to Box and Cox transformation and a robust algorithm that determined the most appropriate weighted mean of the data [13,14]. The RI estimated by nonparametric method was defined based on the 2.5th and 97.5th percentiles with a nonparametric estimation of 90% CI for percentiles. The 90% Cis for lower and upper limits of RI estimated by the robust and nonparametric method were calculated by the bias-corrected and accelerated bootstrap method (BCa) with 5000 bootstrapped samples.

All statistical data analysis was performed in R software, version 4.1.3 [15].

## 3. Results

### 3.1. Sample Characteristics

At the cardiology clinic, a number of 127 full-term newborns were examined to be included in the study, of which two newborns were diagnosed with wide atrial septal defect with significant left-right shunt and one newborn with coarctation of the aorta, thus being excluded from the study group. In addition, in 21 cases, the obtained apical four-chamber view images were unsatisfactory (poor-quality 2D images with foreshortened apex of LV or RV significant lung artifacts obscuring the apex of one or both ventricles, or the walls), and therefore, these cases were also excluded from the study.

The demographic clinical characteristics of the studied sample in the present observational study are summarized in Table 1. The median (IQR) age of the study newborns was 3 (2, 4) days, with a value range of 1–23 days, and 59 (57.28%) newborns were boys.

### 3.2. Reproducibility Analysis of Speckle-Tracking Parameters

The reproducibility analysis for speckle-tracking strain measurements is summarized in Table 2. The degree of agreement between strain measurements of the two observers ranged from good to excellent for all speckle-tracking parameters except for the strain measured on the medial portion of the left ventricle lateral wall and the strain measured on the basal portion of the inter-ventricular septum, which reflected a fair interobserver level of reproducibility (ICC = 0.52, 95% IC: 0.22–0.72 and ICC = 0.43, 95% IC: 0.12–0.67).

**Figure 2:**
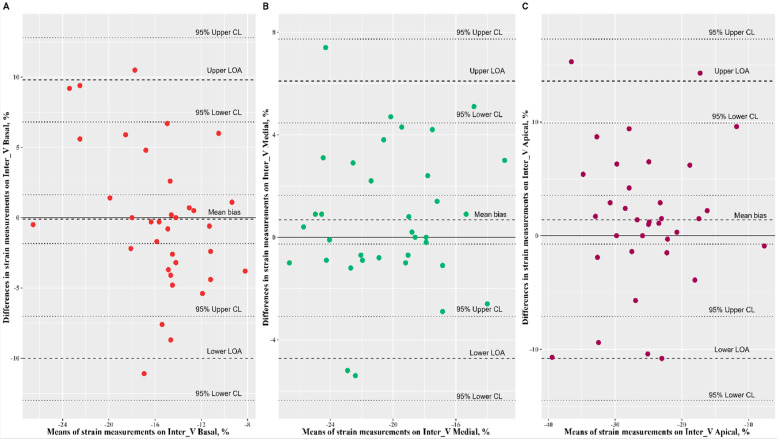
Bland–Altman plots comparing the speckle-tracking cardiac measurements on inter-ventricular septum; (**A**: inter V basal) mean bias: −0.10, 95% CI: (−1.84, 1.63) represented by dotted line, limits of agreement (LOA): −10.01 to 9.80, 95% CI for lower limit: (−13.01, −7.01), 95% CI for upper limit: (6.81, 12.80); (**B**: inter V medial) mean bias: 0.68, 95% CI: (−0.27, 1.63), limits of agreement (LOA): −4.73 to 6.10, 95% CI for lower limit: (−6.37, −3.09), 95% CI for upper limit: (4.46, 7.73); (**C**: inter V apical) mean bias: 1.40, 95% CI: (−0.74, 3.54), limits of agreement (LOA): −10.80 to 13.60; 95% CI for lower: (−14.49, −7.11), 95% CI for upper: (9.91, 17.29).

**Figure 3:**
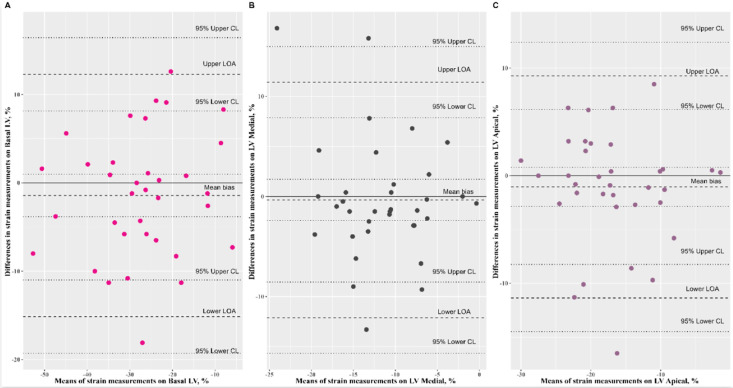
Bland–Altman plots comparing the speckle-tracking cardiac measurements on left ventricle; (**A**: LV basal) mean bias: −1.43, 95% CI: (−3.83, 0.98) represented by dotted line, limits of agreement (LOA): −15.14 to 12.29, 95% CI for lower: (−19.30, −10.99); 95% CI for upper: (8.14, 16.44); (**B**: LV medial) mean bias: −0.34, 95% CI: (−2.40, 1.72), limits of agreement (LOA): −12.11 to 11.42, 95% CI for lower: (−15.66, −8.55); 95% CI for upper: (7.86, 14.98); (**C**: LV apical) mean bias: −1.05, 95% CI: (−2.84, 0.76), limits of agreement (LOA): −11.36 to 9.27, 95% CI for lower: (−14.48, −8.24); 95% CI for upper: (6.15, 12.39).

**Figure 4:**
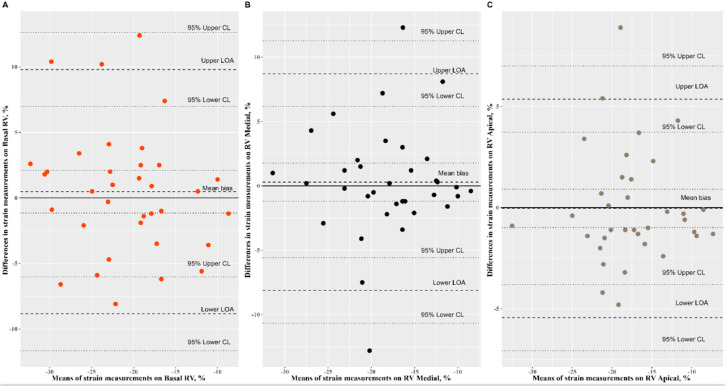
Bland–Altman plots comparing the speckle-tracking cardiac measurements on right ventricle. (**A**: RV basal) mean bias: 0.48, 95% CI: (−1.16, 2.11) represented by dotted line, limits of agreement (LOA): −8.84 to 9.80, 95% CI for lower limit: (−11.66, −6.02); 95% CI for upper limit: (6.98, 12.62); (**B**: RV medial) mean bias: 0.29, 95% CI: (−1.18, 1.77), limits of agreement (LOA): −8.13 to 8.71, 95% CI for lower: (−10.68, −5.58); 95% CI for upper: (6.17, 11.26); (**C**: RV apical) mean bias: −0.04, 95% CI: (−0.98, 0.91), limits of agreement (LOA): −5.44 to 5.36, 95% CI for lower: (−7.07, −3.80), 95% CI for upper: (3.73, 6.99).

The Bland–Altman analysis of speckle-tracking strain measurements yielded a mean bias comprised between −1.43% and 1.40%. The Passing–Bablok regression revealed a nonsignificant systematic bias and nonsignificant proportional bias for all speckle-tracking strain measurements (Figure 2, Figure 3, and Figure 4, Table 2).

### 3.3. Speckle-Tracking Strain Distribution

All speckle-tracking-based strain measurements showed a normal distribution (Table 3), while significant Shapiro–Wilk test results confirmed violations of normality law for strain measured on the basal portion of the inter-ventricular septum (*p*-value = 0.000016), strain measured on the apical portion of the inter-ventricular septum (*p*-value = 0.017), and peak longitudinal strain of the left ventricle (*p*-value = 0.002).

### 3.4. Speckle-Tracking Cardiac Parameters in Relation with Age, Gestational Age, Gender, and Weight

We found no significant correlations of speckle-tracking characteristics with age and gestational age (range for Spearman’s rho correlations values: −0.14 to 0.10 for age and −0.13 to 0.13 for gestational age). Regional and peak longitudinal strain values did not linearly correlate with birth weight except for LV basal, for which we found a weak linear correlation (r = −0.23, *p* = 0.0179). Boys and girls displayed similar distributions of regional and peak longitudinal strain values (*p* > 0.05).

There was no significant difference in peak longitudinal strain values among diagnosis (*p* > 0.05) except for LVpGLS (Kruskal–Wallis test, *p* = 0.042), and the results of post hoc analysis revealed a significant difference between newborns with PFO and those with small muscular VSD (Dunn’s test, *p* = 0.045, median (IQR): −20.70, (−22.70, −18.20) for newborns with PFO vs. −18.35, (−18.65, −17.20) for newborns with small muscular VSD), which became non-significant after adjustment for multiple comparisons (Dunn’s test, adjusted *p*-value = 0.135).

### 3.5. Reference Interval Estimation

**Figure 5:**
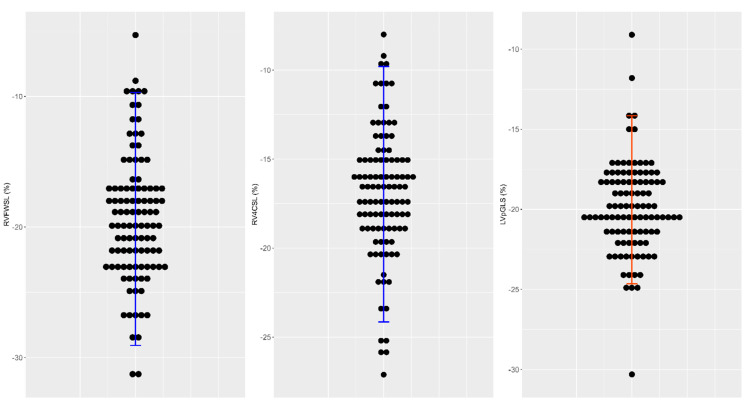
Dot plots with error bars showing the measured values of LVpGLS, RVFWSL, and RV4CSL parameters (data points without outlier removal). The error bars in blue represent mean +/− 1.96 SD, while error bars in red represent the 2.5%th and 97.5% percentiles.

The reproducibility of RI obtained by the parametric method after outlier removal was slightly shorter than those obtained by robust method (Table 4). The same results were observed for the RI obtained by the non-parametric method after outliers were removed (LV pGLS: −24.65, 90% CI: −25.21 to −24.10 and −14.62, 90% CI: −15.14 to −12.24, InterV_Basal: −24.65, 90% CI: −29.83 to −26.29 and −7.41, 90% CI: −8.31 to −6.38, InterV_Apical: −37.48, 90% CI: −40.44 to −35.85 and −15.37, 90% CI: −16.44 to −12.94). The reference intervals obtained for the LV myocardial function LVpGLS, and those for the RV RVFWSL and RV4CSL were represented in Figure 5.

## 4. Discussion

The heart of newborns is distinguished from that of other age categories by multiple characteristics, such as hemodynamic conditions, contractile, and electrochemical properties [4]. The myocardium of the two ventricles differentiates qualitatively and quantitatively throughout the growth process. When the physiology of the fetal circulation changes at birth, respectively, the gas exchange is transferred from the placenta to the lungs, the fetal shunts are gradually closed, and significant hemodynamic changes occur. Neonatal cardiac compliance increases significantly in the first days after birth due to changes at the level of the connective tissue, collagen, and extracellular matrix. Myocardial performance in newborns is influenced by changes in preload, contractility, and afterload. The parasympathetic dominance and immature sympathetic innervations restrict cardiac contractility in newborns [16]. The classical echocardiographic parameters, more precisely the EF, TAPSE, and MAPSE, fail to capture these changes, their values being similar to those of the other age groups. A careful analysis of the longitudinal strain parameters can bring valuable information applicable to the neonatal age group, useful especially in the presence of a certain pathology that requires therapy and monitoring.

Because the data available so far in the literature are limited, and many results found in retrospective studies leave room for prospective studies, the first objective of this study was to test the reproducibility of longitudinal and regional strain measurements and if the acquisition of the four-chamber view images for 2D speckle analysis is made in adequate technical conditions, and the analysis performed was performed by experienced operators. As seen from the obtained results, the measurements are comparable and in agreement. For the LVpGLS, RVFWSL, and RV4CSL parameters, there was an excellent concordance. This prospect is important in encouraging the use of this method in clinical practice, an aspect that is different from other studies. Lorch et al. attributed an important interobserver variability to the learning curve [17]. An important intraobserver and interobserver variability for RV resulted from the study published by Levy and collaborators [6]. In contrast, the study published by Levy et al. demonstrated good clinical feasibility and reproducibility for longitudinal strain parameters for the RV in preterm infants [6].

According to data found in the literature, amongst the most common variables that may influence strain measurements are the following: demographics (age, sex, and ethnicity), clinical parameters (heart rate, weight, body surface area, blood pressure, and LV volumes, size, and mass) [2,7]. In our study, we did not observe variability of peak longitudinal and segmental strain values with demographic parameters, more precisely gestational age, age at birth, sex, and weight. This may be due to the fact that we analyzed only healthy newborns, while several studies from the literature expand the age of the subjects up to 1 year [18]. Although neonates with PFO, ASD, small VSD, and malformed aortic valve are considered to have a heart disease without hemodynamic impact and were found eligible for the determination of normal values, the groups were statistically and individually analyzed, but no statistically significant difference was found between the groups, only a difference with a tendency towards significance for the LVpGLS parameter between the PFO group and small VSD groups.

Analyzing the normal range values obtained in our study, we observed wider reference intervals for the segmental longitudinal strain, with higher values for the basal segments at the ventricular wall level when compared to the apical segments. On the other hand, at the level of the interventricular septum, this aspect was not characteristic, with the apical segment being the one with higher longitudinal strain values.

The reference values for peak myocardial function are much narrower; for the LV, LVpGLS is (−24.65 to −14.62), and for the RV, RVFWSL is (−28.69 to −10.68) and RV4CSL (−22.30 to −11.37).

Normative data and reference intervals are rare and still under development for strain parameters obtained with the help of 2D STE in premature and full-term newborns. Studies in the literature include a relatively small number of newborns [9]. Despite this, we compared our results with data from the literature. Most studies that determine strain values in newborns and infants show a decrease of segmental strain values from the basal towards the apical segments, an aspect that was observed in our study as well [16,19]. In the study of Schubert et al., the authors reported longitudinal strain values measured in the fetal and neonatal period in 30 fetuses and healthy newborns, and concluded that the speckle-tracking method is feasible both in fetuses and newborns [19]. The maximum and average values obtained in the previous study fall within the reference ranges obtained in our study while maintaining higher values for basal segments. Moreover, in the study of these authors, the segmental strain values for RV were higher compared to those determined on LV. Extrapolating this aspect to our results, we observed that RVFWSL, a parameter that analyzes the longitudinal movement of the RV free wall, had the highest absolute value.

Another study on LV function in the newborn was performed by Klitsie et al.: a prospective study conducted on a number of 28 healthy newborns. The mean value of LVpGLS was reported as a value that falls within the reference range of the values reported in our study [20].

In addition, technical variables, such as software or transducer frequency, play an important role in influencing myocardial deformation measurements [2]. Although the echocardiographic evaluation was performed with an Epiq echocardiograph and the speckle-tracking analysis with Qlab 15 software, which is different from existing studies in the literature at present, the reported values are within the reference ranges determined by this study [19,20].

The transition from fetal to neonatal period involves a number of complex hemodynamic changes. We believe that the fetal myocardial immaturity and the cardiopulmonary transition from fetal to neonatal life may be explanatory for the wider range of obtained reference values for the RV compared to those of the LV. The RV strain values reported in our study are comparable to fetal strain value data found in the studies of other authors [21,22].

## 5. Limitations

The present results should be considered within the context of the following potential limitations: (1) Our speckle-tracking-based strain measurements might be biased by the level of experience of the cardiologists, and slightly different results will likely be observed across cardiologists with different levels of experience. However, we tried to account for this possibility by reporting 90% confidence intervals around the lower and upper limits of the reference intervals. (2) The relatively small sample size of our study, which was selected in a single center, cannot, therefore, be generalized to the entire healthy newborn population, and hence, a future multicenter study with a larger sample size is necessary to ensure external validity of the results. However, our healthy newborns were rigorously selected and can be considered as a reasonable sample for a single-center study. In order to account for this issue, we used the different approaches for determination of RI adapted for small sample size. (3) A significant number of newborns was excluded from study due to the poor quality of speckle-tracking images, which could lead to a potential selection bias. (4) Our study lacked the evaluation of intra-observer reproducibility. (5) Another limitation is the heterogeneous distribution of subjects in relation to age, with the neonatal age range being from 1 to 28 days in our study. Although we found no significant correlation between age values and longitudinal and regional strain measurements, future studies should retest the reproducibility feature and reference intervals according to the age groups. (6) The number of newborns with small VSD was very small, so it could not be concluded whether a small left-right shunt with a minimal influence on preload increase could influence LV longitudinal strain values. Future studies should test the potential impact of VSD on strain measurements. (7) We performed peak longitudinal strain of LV measured from a four-chamber view as an estimate of global longitudinal strain of LV due to technical restrictions of speckle-tracking echography in newborns. In this age category, the global longitudinal strain of LV it is not identical to that defined in the adult population.

Despite the previous limitations, the clinical significance of the present study lies in the relatively easy, noninvasive acquisition of echocardiographic images required for 2D STE, an analysis that gives us important information about ventricular function. We consider the parameters for the RV to be of major importance, as they represent the only quantifiable parameters through the echocardiographic method. Knowing the reference values for healthy newborns, this method can be used in the case of newborns with pathology, such as heart malformations, pulmonary hypertension, and heart failure of various etiologies. New prospective studies are needed in order to help demonstrate the exact changes in these parameters depending on the pathology.

## 6. Conclusions

The 2D speckle-tracking method is a reproducible method for the cardiac assessment of healthy, full-term newborns. Two-dimensional peak longitudinal LV and RV strains are reproducible with good to excellent agreement and may represent a possible alternative for the cardiac assessment of healthy newborns in the clinical practice. For assessment of cardiac function, both parameters can be used (LVpGLS to assess LV function and the RVFWSL and RV4CSL for the RV function, respectively). The reference values obtained for the LV myocardial function LVpGLS are between −24.65 and −14.62, and those for the RV RVFWSL are from −28.69 to −10.68 and for RV4CSL are from −22.30 to −11.37.

## Figures and Tables

**Table 1:** Demographic, clinical, and echocardiographic characteristics of studied sample.

Neonates Characteristics	*n* = 103
Age (days), median (IQR)	3 (2, 4)
Gestational age (weeks)	39 (38, 40)
Gender, *n* (%)	
Boy	59 (57.28)
Girl	44 (42.72)
Head_Circumference (cm)	34.50 (33.50, 35.00)
Birth_Length (cm)	54.20 ± 2.70
Birth._Weight (g)	3431.75 ± 467.50
Apgar Score 1 min	9 (9, 10)
Apgar Score 5 min	10 (10, 10)
C-section (yes)	22 (21.36)
Diagnosis	
Patients diagnosed with PFO )	35 (33.98)
Patients diagnosed with small ostium secundum ASD	52 (50.49)
Patients diagnosed with small muscular VSD	4 (3.88)
Patients diagnosed with Bicuspid Aortic Valve	12 (11.65)
Systolic blood pressure (mmHg)	72 (65, 76)
Diastolic blood pressure (mmHg)	37 (35, 45)
Heart rate (beats per minute)	134 (128, 138)
Echocardiographic parameters	
EF (%)	68.73 ± 7.90
TAPSE (mm)	9.06 ± 1.76
MAPSE (mm)	6.80 (6.05, 7.95)

*n*, number of cases; IQR, interquartile interval (lower limit, upper limit); Apgar score at 1 min, Apgar score at 1 min after delivery; Apgar score at 5 min, Apgar score at 5 min after delivery; C-section, Cesarean delivery; PFO, patent foramen ovale; ASD, atrial septal defect; VSD, ventricular septal defect, EF, ejection fraction; TAPSE, tricuspid annular plane systolic excursion; MAPSE, mitral annular plane systolic excursion.

**Table 2:** Interobserver agreement analysis.

Echocardiographic Variables	Observer 1	Observer 2	Interobserver Reproducibility (*n* = 35)			
	Mean ± SD	Mean ± SD	ICC (95% CI)	Systematic Bias (95% CI) Bland–Altman	Intercept (95% CI) Passing–Bablock	Slope (95% CI) Passing–Bablock
LV basal (%)	−28.01 ± 12.44	−26.59 ± 11.54	0.83 (0.69, 0.91)	−1.43 (−3.83, 0.98)	0.13 (−3.98, 3.78)	0.98 (0.75, 1.23)
LV medial (%)	−11.72 ± 5.58	−11.38 ± 6.51	0.52 (0.22, 0.72)	−0.34 (−2.40, 1.72)	1.30 (−1.50, 5.81)	1.00 (0.75, 1.38)
LV apical (%)	−17.36 ± 6.83	−16.31 ± 7.26	0.72 (0.51, 0.85)	−1.05 (−2.85, 0.76)	2.44 (−0.70, 8.25)	1.11 (0.92, 1.43)
Inter V basal (%)	−15.60 ± 3.82	−15.50 ± 5.48	0.43 (0.12, 0.67)	−0.10 (−1.84, 1.63)	10.25 (0.01, 28.43)	1.63 (0.98, 2.79)
Inter V medial (%)	−19.93 ± 3.88	−20.62 ± 3.66	0.73 (0.52, 0.85)	0.68 (−0.27, 1.63)	−1.41 (−6.31, 4.09)	0.94 (0.70, 1.22)
Inter V apical (%)	−24.68 ± 7.75	−26.08 ± 7.30	0.65 (0.42, 0.81)	1.40 (−0.74, 3.54)	−0.90 (−8.04, 8.46)	1.02 (0.71, 1.40)
RV basal (%)	−20.67 ± 6.21	−21.15 ± 6.95	0.74 (0.55, 0.86)	0.48 (−1.16, 2.11)	2.03 (−2.65, 9.62)	1.13 (0.88, 1.47)
RV medial (%)	−17.93 ± 5.89	−18.22 ± 5.76	0.73 (0.53, 0.86)	0.29 (−1.18, 1.77)	0.68 (−2.85, 3.76)	1.03 (0.82, 1.22)
RV apical (%)	−17.50 ± 5.41	−17.46 ± 5.44	0.87 (0.77, 0.94)	−0.04 (−0.98, 0.91)	0.62 (−1.60, 2.94)	0.99 (0.86, 1.15)
LV pGLS (%)	−19.08 ± 3.66	−18.94 ± 3.32	0.70 (0.48, 0.84)	−0.14 (−1.08, 0.80)	−1.65 (−6.30, 8.34)	0.90 (0.66, 1.42)
RVFWSL (%)	−19.01 ± 5.67	−19.26 ± 5.67	0.78 (0.61, 0.88)	0.24 (−1.08, 1.56)	0.85 (−2.96, 5.26)	1.04 (0.84, 1.28)
RV4CSL (%)	−16.52 ± 3.99	−16.34 ± 3.79	0.79 (0.62, 0.89)	−0.18 (−1.06, 0.70)	0.13 (−3.98, 3.78)	0.98 (0.75, 1.23)

SD, sample standard deviation; ICC, intraclass correlation coefficient; bias, mean of the differences; 95% CI, 95% confidence interval; LV, left ventricle; Inter V, interventricular septum; RV, right ventricle; LVpGLS, left ventricle peak longitudinal strain; RVFWSL, right ventricle free wall strain longitudinal; RV4SLC, right ventricle four-chamber strain.

**Table 3:** Distributions of speckle-tracking-based regional and peak longitudinal strain measurements.

Measurements	Descriptive Statistics			
	Mean (SD)	Range (Min, Max)	Median (IQR)	Distribution
LV basal (%)	−29.43 (10.34)	−56.70 to −3.90	−28.80 (−36.00, −22.90)	Gaussian
LV medial (%)	−12.17 (5.19)	−23.50 to −0.70	−12.10 (−16.00, −8.85)	Gaussian
LV apical (%)	−18.72 (7.15)	−35.30 to −1.30	−18.50 (−23.25, −14.20)	Gaussian
Inter V basal (%)	−15.28 (5.23)	−40.40 to −5.60	−14.70 (−17.90, −12.10)	nonGaussian
Inter V medial (%)	−19.57 (3.37)	−27.30 to −11.40	−19.30 (−21.85, −17.45)	Gaussian
Inter V apical (%)	−26.66 (6.61)	−45.90 to −7.00	−26.50 (−30.05, −22.95)	nonGaussian
RV basal (%)	−21.07 (6.06)	−40.02 to −0.10	−21.10 (−24.50, −18.10)	Gaussian
RV medial (%)	−18.18 (5.06)	−30.09 to −7.1	−17.70 (−21.55, −16.05)	Gaussian
RV apical (%)	−17.89 (4.64)	−33.00 to −0.20	−18.40 (−21.05, −14.65)	Gaussian
LV pGLS (%)	−19.85 (2.87)	−30.30 to −9.10	−20.10 (−21.50, −18.10)	nonGaussian
RVFWSL (%)	−19.38 (4.94)	−31.50 to −5.30	−19.60 (−22.80, −17.10)	Gaussian
RV4CSL (%)	−16.97 (3.66)	−27.10 to −8.00	−16.70 (−18.80, −15.00)	Gaussian

SD, sample standard deviation; IQR, interquartile interval; LV, left ventricle; Inter V, interventricular septum; RV, right ventricle; LVpGLS, left ventricle peak longitudinal strain; RVFWSL, right ventricle free wall strain longitudinal; RV4SLC, right ventricle four-chamber strain.

**Table 4:** Reference intervals (RIs) for speckle-tracking-based strain measurements.

Measurements	RI Type	95% RI with Outlier Removal			95% RI without Outlier Removal		
		RI (LL–UL)	90% CI for LL	90% CI for UL	RI (LL–UL)	90% CI for LL	90% CI for UL
LV basal (%) ^(a)^	Parametric	−48.53 to −10.30	−51.26 to −45.80	−13.03 to −7.57	−49.70 to −9.16	−52.56 to −46.83	−12.03 to −6.30
	Robust	−48.66 to −9.66	−51.81 to −45.92	−12.33 to −7.06	−49.79 to −8.47	−53.09 to −46.93	−11.24 to −5.41
LV medial (%)	Parametric	Na	Na	Na	−22.34 to −1.99	−23.78 to −20.90	−3.43 to −0.56
	Robust	Na	Na	Na	−22.58 to −1.87	−23.98 to −21.26	−3.16 to −0.35
LV apical (%)	Parametric	Na	Na	Na	−32.74 to −4.70	−34.72 to −30.76	−6.68 to −2.72
	Robust	Na	Na	Na	−33.09 to −4.54	−35.21 to −31.19	−6.32 to −2.43
Inter V basal (%) ^(b)^	Nonparametric	−26.90 to −7.41	−29.83 to −26.29	−8.31 to −6.38	−27.20 to −6.42	−29.88 to −14.00	−7.24 to −5.16
Inter V medial (%)	Parametric	Na	Na	Na	−26.17 to −12.98	−27.10 to −25.24	−13.91 to −12.98
	Robust	Na	Na	Na	−26.30 to −12.83	−27.34 to −25.35	−13.69 to −12.83
Inter V apical (%)	Nonparametric	−37.48 to −15.37	−40.44 to −35.85	−16.44 to −12.94	−44.26 to −9.30	−51.12 to −42.62	−11.60 to −2.96
RV basal (%) ^(b)^	Parametric	−31.58 to −10.87	−33.06 to −30.09	−12.35 to −9.38	−32.95 to −9.20	−34.62 to −31.27	−10.88 to −7.52
	Robust	−31.81 to −10.73	−33.40 to −30.43	−12.12 to −9.06	−33.30 to −9.16	−35.67 to −31.66	−10.87 to −6.77
RV medial (%) ^(c)^	Parametric	−27.25 to −9.93	−28.51 to −25.99	−11.19 to −8.67	−32.95 to −9.20	−34.62 to −31.27	−10.88 to −7.52
	Robust	−27.33 to −9.47	−28.82 to −25.95	−10.65 to −8.19	−28.30 to −8.02	−29.90 to −26.96	−9.38 to −6.48
RV apical (%) ^(d)^	Parametric	−26.39 to −9.10	−27.62 to −25.16	−10.33 to −7.87	−26.99 to −8.80	−28.27 to −25.70	−10.08 to −7.51
	Robust	−27.73 to −9.03	−27.93 to −25.67	−10.23 to −7.56	−27.21 to −8.60	−28.74 to −26.07	−9.88 to −6.99
LV pGLS (%) ^(b)^	Nonparametric	−24.65 to −14.62	−25.21 to −24.10	−15.14 to −12.24	−24.90 to −13.18	−25.62 to −19.50	−17.26 to −10.48
RVFWSL (%) ^(b)^	Parametric	−28.11 to −10.45	−29.38 to −26.85	−11.72 to −9.18	−29.06 to −9.69	−30.43 to −27.70	−11.06 to −8.32
	Robust	−28.69 to −10.68	−30.09 to −27.64	−11.99 to −9.85	−29.57 to −8.60	−31.23 to −28.37	−11.21 to −8.17
RV4CSL (%) ^(e)^	Parametric	−22.30 to −11.37	−23.11 to −21.49	−12.18 to −10.57	−24.15 to −9.80	−25.17 to −23.14	−10.81 to −8.78
	Robust	−22.45 to −11.31	−23.35 to −21.70	−12.09 to −10.42	−24.13 to −9.50	−25.37 to −23.16	−10.52 to −8.25

RI, reference interval; CI, confidence interval; Cis for Robust method was estimated by bias-corrected and accelerated bootstrap algorithm (BCa); LL, lower reference limit; UL, upper reference limit. ^(a)^ 2 (1.94%) outliers determined by Horn’s method; percentile 2.5−percentile 97.5; outliers detection; ^(b)^ 3 (2.91%) outliers determined by Horn’s method; ^(c)^ 6 (5.82%) outliers; ^(d)^ 1 (0.97%) outliers; ^(e)^ 9 (8.74%) outliers; Na, not applicable because no outliers were found; LV, left ventricle; Inter V, interventricular septum; RV, right ventricle; LV pGLS, left ventricle peak longitudinal strain; RVFWSL, right ventricle free wall strain longitudinal; RV4SLC, right ventricle four-chamber strain.

## Data Availability

The raw data presented in this study can be obtained upon reasonable request addressed to Liliana Gozar lili_gozar@yahoo.com.
